# Dendritic Core-Multishell Nanocarriers in Murine Models of Healthy and Atopic Skin

**DOI:** 10.1186/s11671-017-1835-0

**Published:** 2017-01-23

**Authors:** Moritz Radbruch, Hannah Pischon, Anja Ostrowski, Pierre Volz, Robert Brodwolf, Falko Neumann, Michael Unbehauen, Burkhard Kleuser, Rainer Haag, Nan Ma, Ulrike Alexiev, Lars Mundhenk, Achim D. Gruber

**Affiliations:** 1 Institute of Veterinary Pathology, Freie Universität Berlin, Robert-von-Ostertag-Str. 15, 14163 Berlin, Germany; 2 Institute of Experimental Physics, Department of Physics, Freie Universität Berlin, Berlin, Germany; 3 Institute of Biomaterial Science and Berlin-Brandenburg Centre for Regenerative Therapies, Helmholtz-Zentrum Geesthacht, Teltow, Germany; 4 Institute of Chemistry and Biochemistry, Organic Chemistry, Freie Universität Berlin, Berlin, Germany; 5 Institute of Nutritional Science, University of Potsdam, Nuthetal, Germany

**Keywords:** CMS, Skin, Topical treatment, Dermal delivery, Atopic dermatitis, Oxazolone, Fluorescence lifetime imaging microscopy, Nanomaterials, Multi-domain nanoparticles, Penetration enhancement

## Abstract

Dendritic hPG-amid-C18-mPEG core-multishell nanocarriers (CMS) represent a novel class of unimolecular micelles that hold great potential as drug transporters, e.g., to facilitate topical therapy in skin diseases. Atopic dermatitis is among the most common inflammatory skin disorders with complex barrier alterations which may affect the efficacy of topical treatment.

Here, we tested the penetration behavior and identified target structures of unloaded CMS after topical administration in healthy mice and in mice with oxazolone-induced atopic dermatitis. We further examined whole body distribution and possible systemic side effects after simulating high dosage dermal penetration by subcutaneous injection.

Following topical administration, CMS accumulated in the stratum corneum without penetration into deeper viable epidermal layers. The same was observed in atopic dermatitis mice, indicating that barrier alterations in atopic dermatitis had no influence on the penetration of CMS. Following subcutaneous injection, CMS were deposited in the regional lymph nodes as well as in liver, spleen, lung, and kidney. However, in vitro toxicity tests, clinical data, and morphometry-assisted histopathological analyses yielded no evidence of any toxic or otherwise adverse local or systemic effects of CMS, nor did they affect the severity or course of atopic dermatitis.

Taken together, CMS accumulate in the stratum corneum in both healthy and inflammatory skin and appear to be highly biocompatible in the mouse even under conditions of atopic dermatitis and thus could potentially serve to create a depot for anti-inflammatory drugs in the skin.

**Electronic supplementary material:**

The online version of this article (doi:10.1186/s11671-017-1835-0) contains supplementary material, which is available to authorized users.

## Background

Topical application of drugs to the skin for direct delivery to the epidermis or dermis is often highly desirable for the treatment of skin diseases. Nanosized drug delivery systems, collectively termed nanocarriers, have recently gained large interest for their potential to optimize this topical delivery. Polymeric dendritic core-multishell nanocarriers were specifically designed for this task and have proven promising in several previous in vitro studies. They consist of a core molecule, such as hyperbranched polyglycerol, to which amphiphilic side chains are covalently bound. The part of the side chains bound to the core, for example an alkane chain, creates a lipophilic inner shell and the outer part, e.g., polyethylene glycol, a hydrophilic outer shell or vice versa [1–4]. Consequently, their architecture effectively renders them a unimolecular equivalent to a micelle or, more correctly, a liposome. The rationale of creating such a molecule is to combine the ability of a micelle to transport both hydrophilic and lipophilic molecules as a universal carrier with the stability of a single molecule that does not disintegrate below a critical micellar concentration [3, 5].

The prototype and most investigated substance of this class of nanocarriers is the hPG-amid-C18-mPEG-core-multishell-nanocarrier, from here on referred to as CMS. Recent in vitro and ex vivo studies have shown that CMS may increase the penetration of cargo compounds through the stratum corneum into the viable epidermis and even into the dermis, potentially even more effectively than the more established class of solid lipid nanoparticles under the same conditions [6–8]. However, studies on the skin penetration behavior of the carrier itself in various in vitro and ex vivo models have yielded controversial results [8] and CMS have not yet been investigated in vivo.

Furthermore, as nanocarriers may be specifically intended to be applied to diseased skin, it is imperative to establish whether alterations of the skin barrier under conditions of disease may change the penetration behavior of the particles [9]. An increased penetration into diseased skin would require a more severe scrutiny of local and systemic biosafety issues. On the other hand, such an effect could be potentially exploited for delivering a larger amount of drug to lesional skin than to the adjacent healthy skin. Indeed, recent in vitro data have suggested that CMS may exhibit increased penetration in several models of altered skin barrier function [6]; however, they have not yet been investigated on diseased skin in vivo.

Atopic dermatitis (AD) is a prime example of a common skin disease with potentially altered skin barrier function that may benefit from nanocarrier formulations. AD is among the most common inflammatory skin diseases in humans worldwide, affecting up to 25% of children and up to 3% of adults [10, 11]. The condition is clinically characterized by highly pruritic, erythematous, and often excoriated plaques [11, 12]. It is a chronic condition and typically requires long-term treatment with topical anti-inflammatory drugs. In AD, epidermal cornification, differentiation, and possibly lipid composition are thought to be altered. The resulting complex disturbances of skin barrier function are still poorly understood, but may allow for enhanced outside-to-inside penetration of antigens [13–17] as well as potentially drugs [18], possibly including nanoparticles under certain conditions [19]. To this date, the penetration of CMS through AD skin has not been investigated in vivo, as is true for most other nanoparticles.

Here, we investigated the penetration of CMS as a prototype of the novel class of dendritic core-multishell nanocarriers into normal mouse skin and a mouse model of AD. The oxazolone-induced mouse model displays structural barrier alterations and clinical signs highly similar to human AD [20]. We hypothesized that CMS would not penetrate into healthy skin but may show increased penetration through the inflamed AD skin. In addition to standard microscopy, we employed a novel approach using fluorescence lifetime imaging microscopy (FLIM) to obtain a more detailed insight into the CMS distribution in the skin and beyond, based on the sensitivity and uniqueness of fluorescence decay curves [21]. Furthermore, local and systemic effects of topical CMS application were examined, complemented by in vitro toxicity assays. Finally, we asked in which organs CMS would be deposited and whether CMS could potentially induce any adverse systemic effects in case of full penetration through a diminished skin barrier by mimicking such a penetration using subcutaneous injection of the full dose that would be typically applied topically.

## Methods

### Particle Synthesis and Characterization

CMS were synthesized and labeled with the fluorescent dye indocarbocyanine (ICC) basically as described earlier [1], with minor modifications, see Additional file 1, resulting in the empirical formula hPG_10000_[-NH_2_-ICC_0.02_(C18-mPEG350)_0.98_]_0.7_ (CMS-ICC). Dynamic light scattering [7] revealed a mean particle diameter of 12.4 ± 3.5 nm.

### Animals and Study Protocol

The study protocol was approved by the State Office of Health and Social Affairs, Berlin, Germany (LaGeSo; G 0126/13) and performed according to national guidelines. All substance applications and measurements of clinical parameters were conducted under isoflurane anesthesia. Mice lay on a heating mat during all procedures.

### Induction of Atopic Dermatitis Model

AD was induced via repeated topical hapten challenges with oxazolone (OX, Sigma-Aldrich, St. Louis, USA) in 6- to 8-weeks-old, male, hairless SKH-1 mice (Charles River, Sulzfeld, Germany) as described [20] with minor modifications. Briefly, mice were sensitized with 5% OX on a 1.5 × 1.5 cm area of the right flank, followed by repeated challenges with 0.1% OX as described in Additional file 2.

### Topical Application of Nanocarriers

CMS-ICC (44.5 μl, 5 g/l) in 0.9% sodium chloride (NaCl) solution or the solvent alone were topically applied twice daily for five consecutive days to the right flank of healthy mice or the inflamed skin area of the AD model (Table 1, Additional file 2: a, b). Topically applied substances were massaged for 3 min and allowed to dry and seep in for 1 h prior to termination of the anesthesia.

**Table 1:** Experimental groups in vivo

Group	Mouse model	Test substance	Route of application
1	Healthy	CMS-ICC (in NaCl solution)	Topically applied
2	Healthy	NaCl-solution	Topically applied
3	Atopic dermatitis	CMS-ICC (in NaCl solution)	Topically applied
4	Atopic dermatitis	NaCl-solution	Topically applied
5	Healthy	CMS-ICC (in NaCl solution)	Subcutaneous injection
6	Healthy	NaCl-solution	Subcutaneous injection

### Subcutaneous Injection of Nanocarriers

To mimic a complete transepidermal uptake, CMS-ICC in 0.9% NaCl solution (150 μl, 5 g/l, i.e., approx. 30 mg/kg body mass) or vehicle only were subcutaneously injected into the right flank of an additional group of SKH-1 mice twice daily for five consecutive days (Table 1, Additional file 2: c).

### Measurement of Clinical Parameters

During the entire experiment, mice were clinically examined and the dermatologic standard parameters of transepidermal water loss (TEWL), erythema index, and skin hydration were quantified prior to each substance application.

### Sacrifice and Sampling

Approximately 16 h after the respective last treatment, animals were sacrificed by isoflurane overdose and tissues were sampled and processed as described [22].

### Particle Localization via Fluorescence Microscopy and Fluorescence Lifetime Imaging Microscopy

**Fig. 1:**
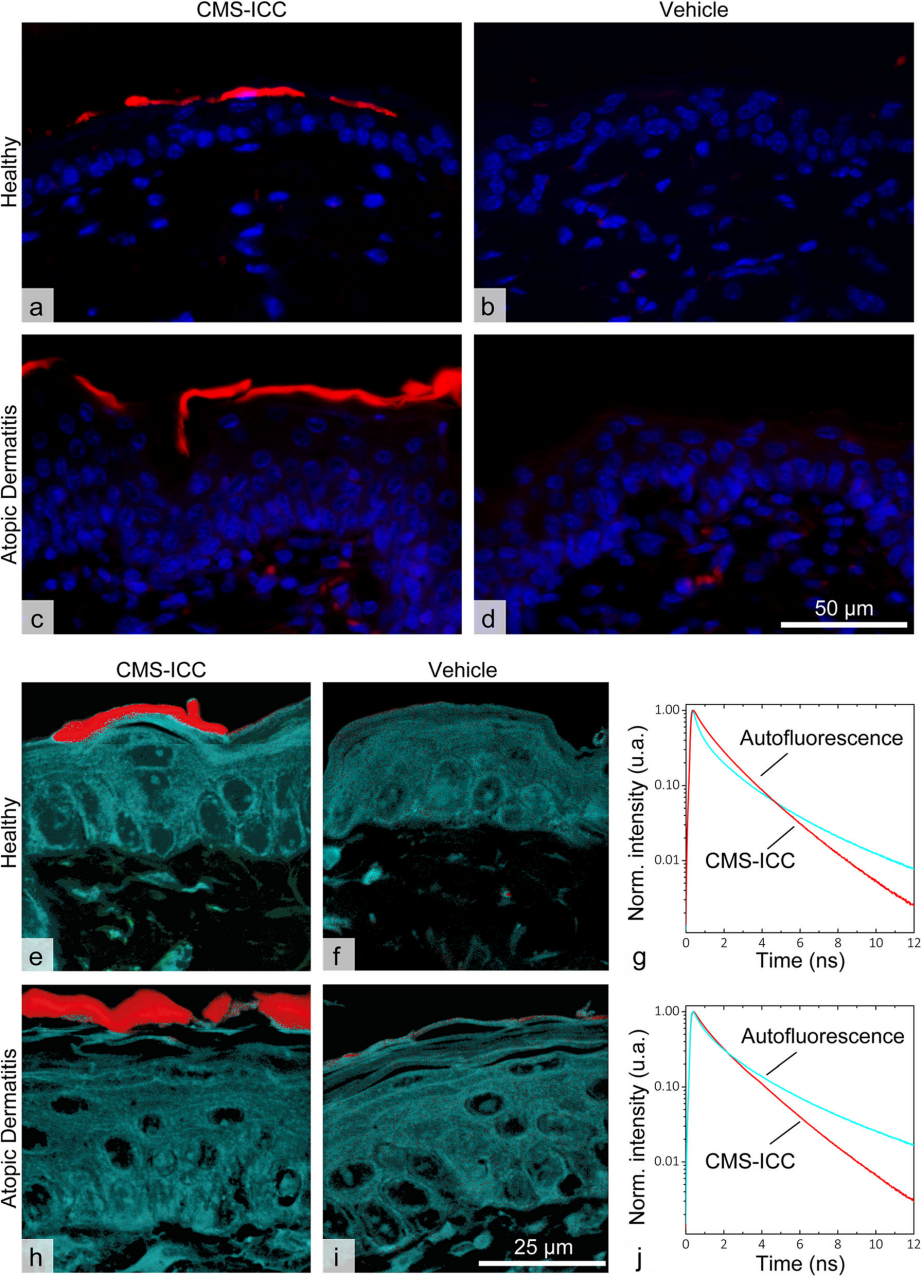
Atopic dermatitis did not influence the dermal penetration of CMS-ICC in mice. **a** ICC-labeled CMS were exclusively found in the stratum corneum of healthy mice. **c** This distribution pattern was virtually identical in mice with atopic dermatitis. **b**, **d** No fluorescent ICC-signals were detected in the skin of the respective control groups. **e**, **h** FLIM analysis confirmed that CMS-ICC exclusively accumulated in the stratum corneum in healthy mice and in mice with atopic dermatitis, based on data analysis of unique fluorescence decay curves (**g**, **j**) from tissue autofluorescence (*cyan*) and from CMS-ICC (*red*). **f**, **i** FLIM images of vehicle-treated skin show tissue-derived autofluorescence (*cyan*). Fluorescence intensities in (**a**–**d**) are adjusted to vehicle control groups and contrast-enhanced post acquisition. False color coding of the FLIM images (**e**, **f**, **h**, **i**) corresponds to the color coding of the fluorescence decay curves (**g**, **j**). *Scale bar* shown in (**i**) refers to images (**e**, **f**, **h**, **i**) *scale bar* in (**d**) to (**a**–**d**)

The distribution of CMS-ICC was evaluated via a fluorescence microscope on formalin-fixed, paraffin-embedded, 4′,6-diamidino-2-phenylindole (DAPI) counterstained sections from all tissues in groups 1, 3, and 5 (Table 1) while the respective vehicle treated groups 2, 4, and 6 served as negative controls as previously described [22]. Additionally, selected sections of the skin (groups 1 to 4) were analyzed by FLIM [21] to confirm the findings from conventional microscopy with increased specificity. In particular, FLIM allows to better differentiate between low level specific signals and autofluorescent tissue background. Increased specificity is based on CMS-ICC exhibiting a fluorescence decay curve that is very distinct from all autofluorescent substances observed in the skin samples (Fig. 1g, j). The setup consisted of an inverted microscope (Olympus IX71) equipped with a ×60 water objective (Olympus UPLSAPO 60XW; NA: 1.2), a confocal laser galvanometer scanning unit (DCS-120, Becker & Hickl, Berlin, Germany), and a picosecond tunable white light supercontinuum laser source in combination with an acousto-optical tunable filter (SuperK Extreme EUV3 and SELECT UV-VIS, NKT Photonics) [23]. Paraffin sections of healthy and atopic murine skin were measured through 0.13–0.15-mm-thick cover glass. CMS-ICC- and vehicle control (NaCl-solution)-treated skin was excited at 530 nm (0.22 mW) with a pulse repetition rate of 19.5 MHz. The resulting fluorescence emission was collected for 900 s and spectrally selected by a long-pass filter (575 LPET, Chroma). The ratio of the stop⁄start pulse rate was normally less than 200:1, appropriate for time correlated single photon counting (TCSPC) [24]. The fluorescence decay traces in each image pixel were detected in 1024 channels with a channel width of 19.5 ps by a hybrid photomultiplier tube (HPM-100-40, Becker & Hickl) and a TCSPC module (SPC-160; Becker & Hickl). The instrument response function of the system was less than 100 ps full width at half maximum. FLIM data were analyzed using self-written routines in C++. Fluorescence decay traces of the individual pixels were partitioned into classes (i.e., clusters) using a multivariate pattern recognition method. False-color images were generated by assigning a distinct color to all pixels containing a fluorescence decay curve that belongs to a certain cluster. To adjust the fluorescence excitation intensity to the different amounts of CMS-ICC fluorescence in the skin, images were taken subsequently for the upper and lower epidermis and analyzed together.

### Histopathologic and Digital-Morphometric Analyses

Mouse organs were examined histopathologically by veterinary pathologists (MR, HP, LM) using formalin-fixed, paraffin-embedded sections stained with hematoxylin and eosin (H&E). Tissues and organs examined included treated skin, untreated skin (contralateral flank), inguinal lymph nodes, axillar lymph nodes, accessory axillar lymph nodes, spleen, liver, lung, brain, thymus, heart, stomach, small and large intestine, mesenterial lymph nodes, pancreas, thyroid glands, adrenal glands, kidneys, testicles, and bone marrow. Relevant dermal inflammatory parameters were quantified via an observer-blinded analysis. The thickness of H&E stained epidermis was measured without the stratum corneum on 66 randomly chosen areas between the hair follicles on scanned slides using digital image analysis software (Image Scope, Aperio Technologies, version 10.2.). Mast cells stained with toluidine blue and eosinophils stained with congo red were counted as described [25]. CD3+ T lymphocytes were labeled immunohistochemically and quantified as described [25].

### In Vitro Toxicological Analyses

Cell viability, cytotoxicity, apoptotic activity, and oxidative stress were analyzed in human immortalized keratinocytes (HaCaT cells) after exposure to 50, 100, 200, 500, or 700 μg/ml CMS via the Cell Counting Kit-8 (CCK-8) assay, the bis-AAF-R110 cell cytotoxicity assay, the caspase 3/7 apoptosis assay, or the H2-DCF-DA assay for reactive oxygen species, respectively, as described in Additional file 1.

### Statistical Analyses

Clinical and histological data are presented as mean ± standard deviation (SD). In vitro toxicological data are presented as mean ± standard error of the mean (SEM). For clinical parameters, day to day differences were calculated to account for different starting values at the beginning of the experiment and analyzed for each day by multiple *t* tests, corrected for multiple comparisons with the Holm-Sidak method (two tailed; H_0_ = “means of day to day differences are the same between CMS and vehicle treated group”). For epidermal thickness, group means were corrected for the respective untreated, contralateral skin because of an existing systematic difference in overall epidermal thickness between the groups. Means of corrected epidermal thickness as well as means of mast cell and lymphocyte counts were then analyzed using Student’s *t* test (two tailed; H_0_ = “means of parameters are the same between CMS and vehicle treated groups”). Eosinophil counts were analyzed using the Kolmogorow-Smirnov two sample test, as Poisson rather than a Gaussian distribution was assumed (two tailed; H_0_ = same as above). For in vitro cytotoxicological assays, means of all CMS concentrations and positive controls were compared to the respective negative controls using Student’s *t* test (one-tailed, H_0_ = “means of concentrations/positive control are the same as the negative control”). For all tests, an alpha error of 5% was accepted.

## Results

### Topically Applied CMS Accumulate in the Stratum Corneum Only

Following topical application over 5 days, both standard fluorescence microscopy and FLIM measurements localized the ICC-labeled CMS nanocarriers exclusively in the stratum corneum of healthy hairless mice (Fig. 1a, b, e, f). CMS did not accumulate in the utriculi/hair follicles.

As shown in Fig. 1g, distinct fluorescence decay curves were extracted from the FLIM data for tissue autofluorescence (cyan curve) and CMS-ICC (red curve) by the analysis algorithm [8]. FLIM images with the corresponding false color coding (Fig. 1e, f) show a clear-cut difference between the ICC-labeled CMS and vehicle treated skin, underscoring the selective and highly sensitive detection of labeled CMS. Thereby, FLIM analysis confirms that the distribution of CMS-ICC is confined to the upper layers of the stratum corneum and that the signals can clearly be distinguished from tissue autofluorescence (Fig. 1e).

### Atopic Dermatitis Does Not Influence CMS Penetration

**Fig. 2:**
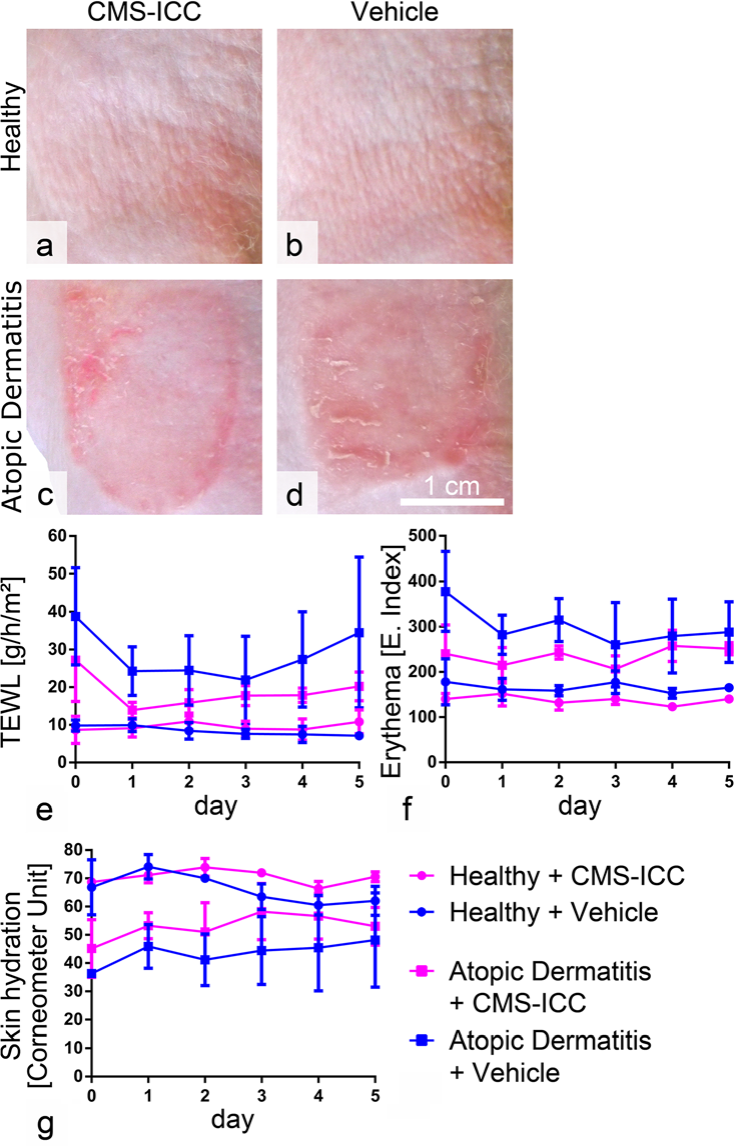
None of the clinical parameters tested were affected by CMS in healthy skin or atopic dermatitis. **a**–**d** Topical application of CMS-ICC had no visual effects on the skin of healthy mice or mice with atopic dermatitis when compared to the solvent-treated controls. **e**–**g** Clinical parameters transepithelial water loss (TEWL), erythema index, and skin hydration were not significantly affected by topically applied CMS-ICC. Note that different starting points have to be taken into account which was addressed statistically by analyzing day to day differences rather than absolute values. Data are presented as mean ± SD, lines connecting data points are visual aids only. No statistical significances were found

**Fig. 3:**
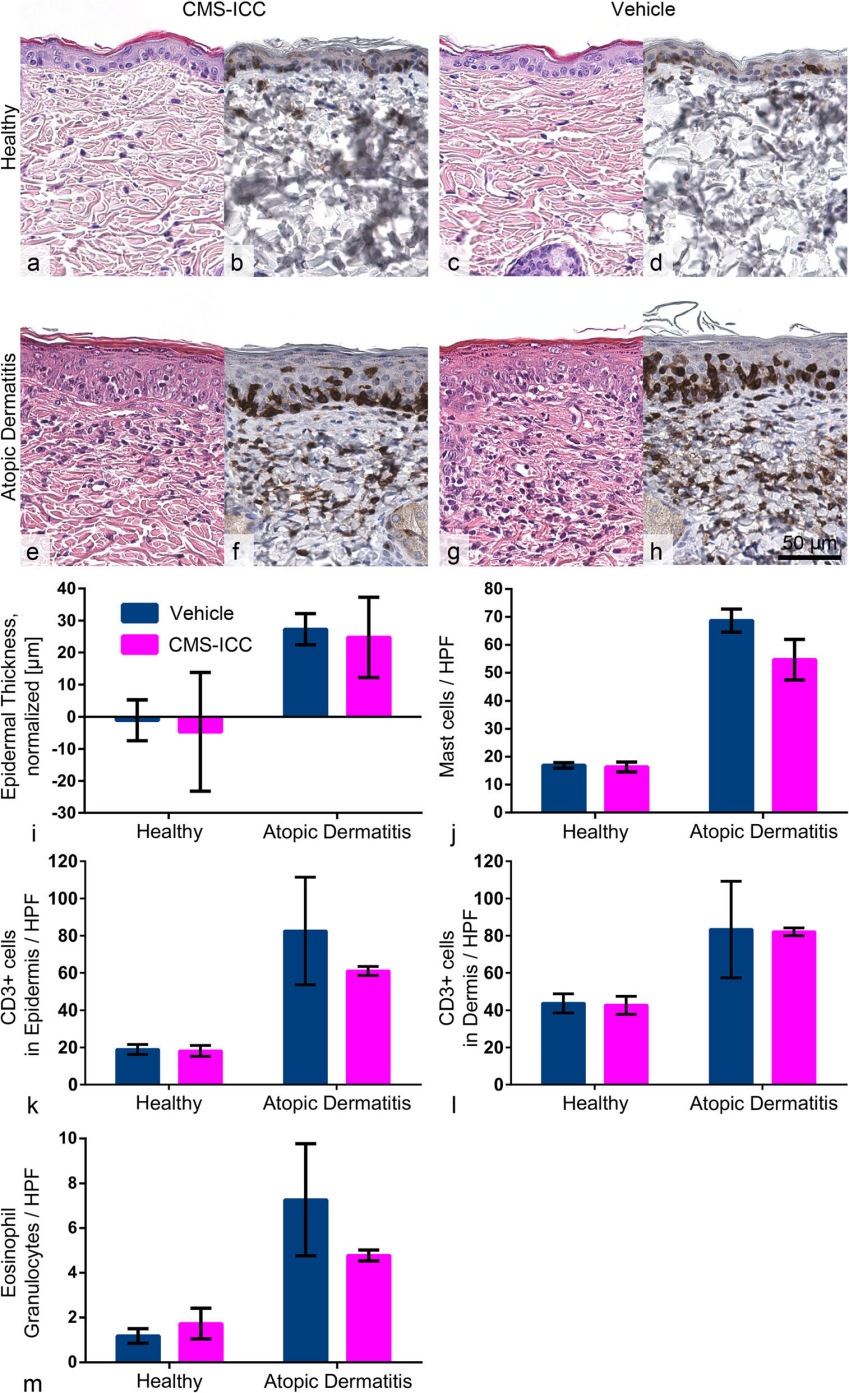
Histological and morphometric analyses failed to reveal any effects of CMS on healthy skin or atopic dermatitis. **a**–**d** Topically applied CMS did not alter the overall histologic appearance (**a**, **c**) or the infiltration with CD3+ T lymphocytes (**b**, **d**) in healthy mice compared to vehicle-treated controls. **e**–**h** In the model of atopic dermatitis, the epidermis was thickened and increased infiltration of immune cells, predominantly T lymphocytes, was observed. However, all parameters tested were unaffected by topically applied CMS when compared to vehicle-treated controls. **i**–**m** Quantification of epidermal thickness corrected for the untreated, contralateral skin, infiltrating mast cells, CD3+ T lymphocytes, and eosinophils revealed no impact of CMS-ICC on healthy skin or the atopic dermatitis model. Data are presented as mean ± SD. HPF = high power field

The signal pattern was identical in mice with oxazolone-induced AD with no evidence of deeper penetration due to inflammation-associated barrier alterations (Fig. 1c, d, h, i). Again, distinct fluorescence decay curves could be extracted (Fig. 1j) and FLIM images with a clear signal to background distinction were generated to exclude overlap with tissue autofluorescence. Nevertheless, clinical data including TEWL, erythema index, and skin hydration as well as histologic parameters clearly confirmed the presence of barrier changes typically associated with AD (Figs. 2 and 3).

### CMS Are Biocompatible In Vivo

Clinical data quantification and statistical analyses failed to reveal any statistically significant differences between CMS-treated and solvent-treated skin in healthy as well as AD groups. This indicates that topically applied CMS had no detectable biological effect on normal mouse skin or the course or severity of inflammation in the AD model (Fig. 2).

Effects of CMS on healthy skin and the phenotype of the AD model were further examined histopathologically. All parameters tested, which included standard histological examination, measurement of epidermal thickness as well as detection and quantification of CD3+ lymphocytes, mast cells, and eosinophils, yielded no evidence of consequences of CMS application when compared to skin treated with vehicle alone (Fig. 3).

### CMS Are Biocompatible In Vitro

**Fig. 4:**
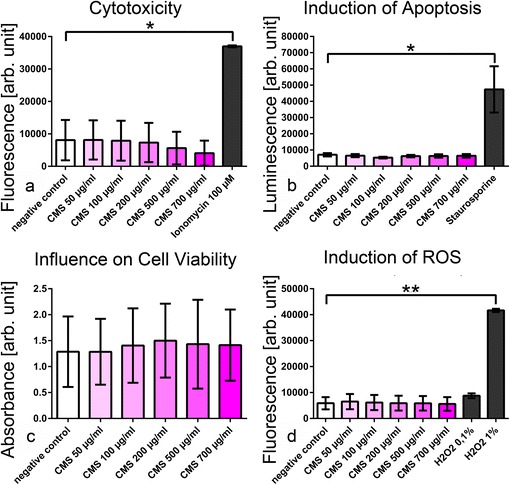
CMS exhibited good biocompatibility in vitro. **a**–**d** CMS concentrations between 50 and 700 μg/ml did not differ significantly from the negative control (untreated cells) in cytotoxicity (bis-AAF-R110 assay), induction of apoptosis (caspase 3/7 assay), cell viability (CCK-8 assay), and production of reactive oxygen species (H_2-_DCF-DA assay). Data are presented as mean ± SEM, *p < 0.05, **p < 0.005

No cytotoxicity of CMS, reduction in cell viability, induction of apoptosis, or oxidative stress on HaCaT cells were observed in all models employed (Fig. 4).

### Mimicking Maximal Penetration of CMS Leads to Systemic MPS Distribution Without Evidence of Toxicity

**Fig. 5:**
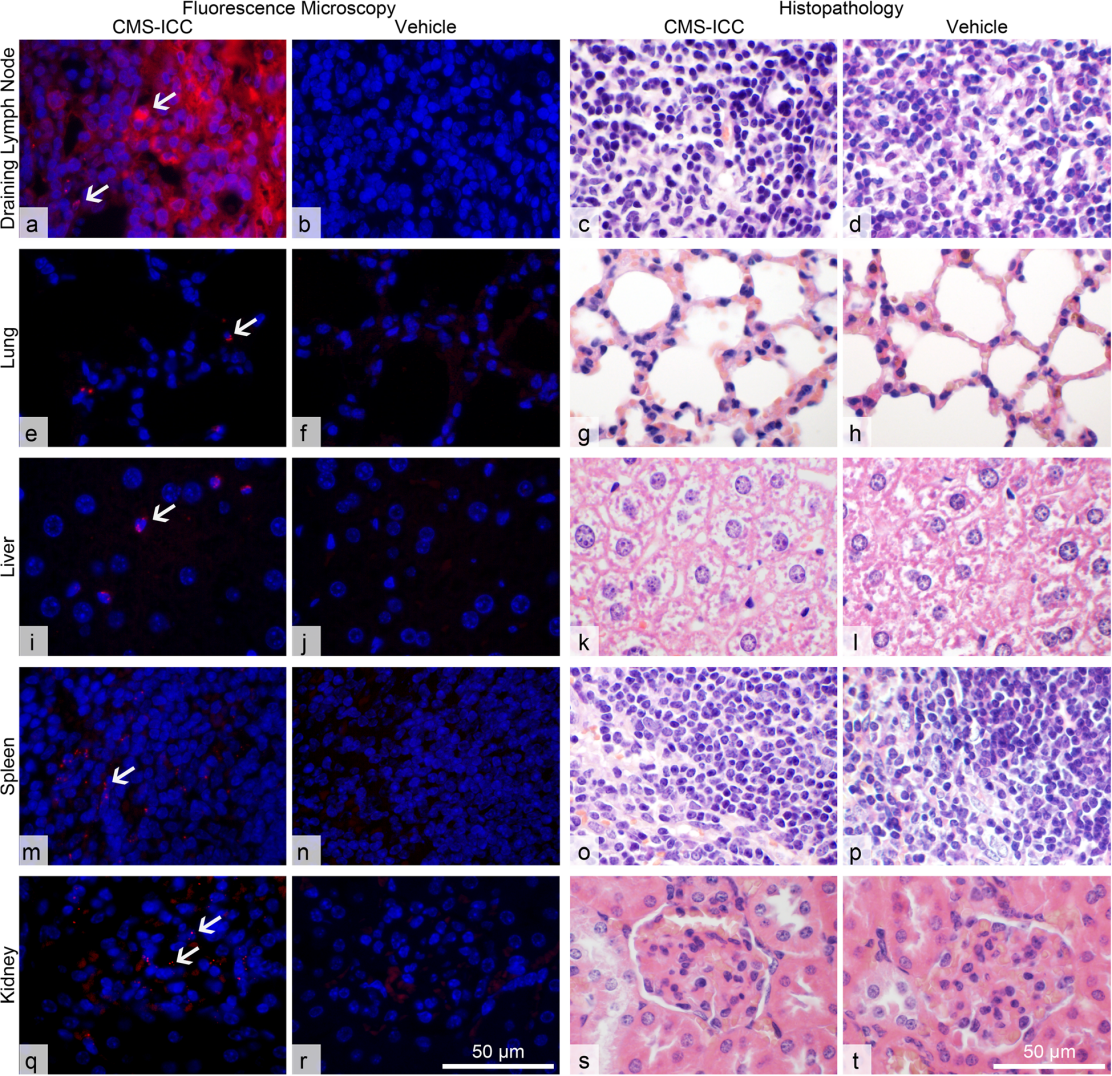
Subcutaneously injected CMS-ICC were localized in the regional lymph nodes, lung, liver, spleen, and kidney of healthy mice without any toxic or other adverse effects. **a**–**t** Approx. 16 h after the last subcutaneous injection, CMS-ICC were predominantly found in and between cells of the draining lymph nodes (**a**, *arrows*). Furthermore, CMS-ICC were found in the alveolar septa of the lung (**e**, *arrow*), intracellularly in cells located within the sinusoids of the liver, consistent with Kupffer cells (**i**, *arrow*), throughout the splenic parenchyma both intracellularly and extracellularly (**m**, *arrow*), and in the kidney, predominantly localized in the glomeruli (**q**, *arrows*).

To mimic a potential transcutaneous uptake and the resulting biodistribution and sequelae for the entire organism, CMS were injected into the subcutaneous tissue of healthy mice with the same time regime used for topical dermal application. In addition to their diffuse deposition at the injection site and the adjacent skin, the particles were found in the local draining lymph nodes and in cells consistent with the mononuclear phagocytic system (MPS) in the liver, spleen, and interalveolar septa of the lung (Fig. 5) approx. 16 h after their last subcutaneous injection, clearly showing that particles entered the systemic circulation. Non-draining lymph nodes remained negative, suggesting that the particles did not reach all lymphatic organs through the circulation. Furthermore, CMS were localized in the glomeruli and occasionally basolaterally to the tubular epithelial cells in the kidney but not in any of the other organs examined.

Histopathological analyses by trained veterinary pathologists (MR, HP, LM) failed to identify any pathologic changes in the potential target organs identified (skin, local lymph nodes, liver, spleen, lung kidney; Fig. 5) or any of the other tissues examined.

## Discussion

Our study makes two main observations: First, it suggests that CMS, when applied topically to murine skin in vivo, accumulate in the stratum corneum but do not surmount this barrier to penetrate into the viable epidermis or beyond. Second, it provides evidence that the complex skin barrier alterations associated with AD do not result in increased penetration or otherwise altered distribution of these nanocarriers.

### Penetration of CMS Topically Applied to Healthy Skin

The local penetration pattern on healthy skin is in accordance with the only previously published results on the penetration of topically applied CMS in vitro, when relatively short incubation times of 6 h on excised human skin or 3 h on reconstructed human skin were used [8]. However, in these models, CMS penetrated into viable skin layers after prolonged incubation times of 24 or 6 h, respectively [8]. It seems likely that overhydration of the skin barrier in the Franz cell setup due to the long contact to the water of the acceptor compartment may have increased permeability in that study and contributed to this divergence. Our study thus adds evidence that incubation times of 6 or 3 h are better suited when using the respective human in vitro systems to replace animal studies for CMS-type carriers.

In addition to the skin being fully viable and in its complete physiological environment, our study adds three important levels of complexity to current knowledge derived from previous in vitro experiments: (a) a fully functional circulatory system, (b) a fully functioning immune system, and (c) physiological flexing and movement of the skin. We were unable to detect labeled CMS in the local lymph nodes or in any other organ which supports our conclusion that they did not surmount the cutaneous barrier even under conditions of physiological tissue fluid—blood/lymph equilibrium. Furthermore, lack of deposition in the local lymph nodes adds evidence that CMS applied topically did not elicit large scale uptake and presentation by Langerhans cells. Langerhans cells are constantly sampling substances from the internal border of the stratum corneum and thus could have potentially come into broad contact with CMS [26, 27]. Finally, there is evidence that mechanical flexing of the skin may enhance nanoparticle penetration [28, 29], an effect not at all addressed by standard in vitro or ex vivo models. Since mice were breathing during the hour of incubation and moving around normally during the rest of the time, physiological movement and stress of the skin was depicted closer to a lifelike application.

In our setting, we were unable to detect any accumulation of CMS in hair follicles/utriculi. Although hair follicle architecture is altered in the SKH-1 mouse, thi﻿s is worth noting as hair follicles have been shown to be an important site of accumulation for certain nanoparticles [30].

No methods were available enabling us to visualize the subcellular niche of the stratum corneum that CMS accumulate in. CMS theoretically possess a hydrophilic outer shell and they thus may be assumed to take the route through the corneocytes. On the other hand, it seems equally possible that they accumulate in the extracellular lipid matrix. In non-polar environments, the outer, hydrophilic groups of CMS have been shown to partially fold inward, exposing the lipophilic inner regions and giving them good solubility in polar solvents similar to a supramolecular micelle reversing its polarity [4]. In fact, the steric arrangement of CMS may altogether be more complicated than the “core-multishell” model traditionally proposed, as the steric arrangement of the polar and non-polar regions may not actually be shell-like. Instead, they may rather form smaller, more randomly distributed domains, which is why the particles possibly are more correctly termed multi-domain nanocarriers [4]. In light of these findings, an intercalation of CMS particles among each other has been proposed [4]. It thus seems plausible to assume that intercalation with the bilayers of the stratum corneum lipid matrix is possible. In addition, CMS are highly flexible and it has been proposed that they may flatten down to a thickness of one nanometer [8]. On the contrary, they may also form larger supramolecular aggregates, depending on the solvent environment and even loaded cargo molecules [2]. In conclusion, there is a multitude of imaginable modes of interaction with the stratum corneum which need further elucidation by functional studies in vitro.

### Penetration of CMS Topically Applied to Atopic Dermatitis

Opposed to our initial hypothesis, the severe morphological and presumably functional barrier alterations associated with AD [13–16, 18, 31] did not increase the penetration of CMS past the stratum corneum. Although this is in accordance with CMS data from a peeling skin disease model in vitro [8], other models previously used to mimic diseased skin have yielded evidence of increased CMS penetration, namely excised human skin subjected to 30 tape strips and an in vitro non-melanoma skin cancer model [8]. However, caution is warranted when comparing the different models as they constitute very different mechanisms of barrier alterations.

So far, CMS have never been investigated in AD patients or in vivo or ex vivo models of AD. While non-particulate substances including water and dyes have been suggested to more effectively penetrate into human AD skin [13–18], data on nanoparticles are scarce. The only other nanoparticles that have been tested in AD patients in terms of altered penetration are zink-oxide particles which also did not surmount the stratum corneum in three human AD volunteers [32]. On the contrary, different zinc-oxide nanoparticles did show increased penetration in a mouse model of AD [19]. Clearly, CMS particles possess a unique architecture with flexible side chains and variable surface properties [4], largely limiting any comparisons of our findings to the penetration behavior of other particles, including zink oxide.

The lack of increased penetration into lesional atopic skin has two practical implications: Most importantly, it yields increased confidence in the biosafety of the particle as there will be no increased contact with viable tissue, at least under the conditions tested here. Such increased penetration into diseased skin is always of concern for nanoparticles [9]. On the other hand, it also means that such an increased penetration cannot be used as a “targeted delivery system” simply by having carrier and the loaded drug preferentially penetrating into lesional skin but not into the adjacent, healthy skin. However, as the penetration enhancement effect of CMS on loaded drugs does not seem to depend on the penetration of the particles themselves [8], it remains to be investigated whether there is an effect of such a barrier alteration on the rate of penetration enhancement by CMS.

### But What About the Penetration Enhancement Effect Shown Previously?

The CMS investigated here have previously been shown to increase the penetration of a loaded model drug into the viable skin past the stratum corneum in vitro [6–8]. Besides making drugs soluble in a variety of solvents in a stable, unimolecular micelle and potentially forming a drug depot in the stratum corneum, this penetration enhancement effect is one of the main benefits sought after in the design of the CMS. However, the aforementioned in vitro experiments already showed that this penetration enhancing effect does not seem to depend on the penetration of the particle itself [8]. While we have not enough data yet to effectively speculate on the mechanism of this penetration enhancement, the particles do contain long, flexible, amphiphilic side chains containing saturated carbon chains, which have been suggested as an ideal chemical structure for penetration enhancement [33]. Intercalation into and modification of the lipid bilayer structure of the stratum corneum extracellular matrix immediately leaps to mind. Clearly, further studies elucidating the underlying mechanism are needed. Nevertheless, whatever the mechanism may be, this property of the particle can be seen as a very welcome one: It has been suggested that one of the great potential benefits of nanocarriers as penetration enhancers may very well consist of their local action at the place where the enhancement is needed most—the stratum corneum—without penetrating themselves, as smaller molecules might [34]. In any case, further studies are needed to establish possible penetration enhancement effects in vivo by CMS for typical anti-inflammatory drugs and a potential therapeutic surplus value, both on healthy and barrier-altered skin.

### Biocompatibility

We also examined potential biological effects of CMS. Both in vitro toxicological data and the systemic distribution suggest acceptable biocompatibility. Nonetheless, this is a preliminary study which was primarily designed to investigate CMS penetration, conducted with a small number of animals for a limited time and thus with limited statistical power in terms of biocompatibility. Moreover, it is important to consider that during topical application small numbers of nanoparticles might reach the dermis and get removed by venous or lymphatic drainage without being detected by the snapshot of microscopic particle detection [9]. The fact that we failed to detect accumulation in the target organs we had identified from the subcutaneous injection gives us a little more confidence that no particles actually penetrated after topical application. Of course, 5 days of application are not nearly sufficient to investigate chronic exposure and potential long time accumulation and that low numbers of particles below the detection limit may have been overlooked. These questions will have to be addressed in further, more comprehensive, and longer-term safety studies.

### General Limitations

A further limitation to this study obviously is the difference between human and murine skin [35]. Nevertheless, it is generally accepted that the human skin is even less penetrable than the murine skin under a variety of circumstances [36]. Thus, our conclusion that CMS do not penetrate beyond the stratum corneum can likely be transferred to human skin, until proven otherwise.

Of equal importance, there is a great potential of differences between the hapten-induced model of AD used here and human AD, the etiology of which is still incompletely understood and might include many additional factors, such as inherited barrier dysfunctions [13, 15, 16, 31, 37]. On the other hand, strong similarities in the barrier alterations of the model used here and human AD have been depicted [20] and our model thus appears as among the most suitable and practicable models available [38].

Another limitation for our conclusions is the question of generalizability of our results to other members of the class of dendritic core-multishell unimolecular micelles. Designs varying in core architecture, alkyl chain length, bond types linking the building blocks, and surface functionalization have been synthesized and are constant subjects to optimization [3, 39–41]. As these particles generally have similar size and distribution of hydrophilic and hydrophilic compartments, however, they are expected to behave similarly in tissues. Clearly, further studies are needed to elucidate the mechanical and biological interaction with the dermal barrier and cells to allow for a better understanding and accurate predictions of the behavior of each particular carrier molecule.

## Conclusions

Our in vivo data show that topically applied unimolecular micelle core-multishell nanocarriers accumulate in the stratum corneum of healthy skin. Importantly, no increased or otherwise altered penetration was observed in a model of atopic dermatitis, a common inflammatory skin disorder with marked barrier disturbances. Together with their ability to enhance penetration of even very large model drugs previously shown in vitro, our observations suggest that CMS may be suitable candidates for the encapsulation of drugs in order to release them from a stratum corneal depot and enhance their penetration into the viable epidermis without penetration of the carrier and thus without biological effects by the carrier itself.

## Additional Files

Additional file 1: Detailed Methods. (DOCX 40.1 kb)
Additional file 2: Schematic Experimental Protocol. (TIFF 916 kb)

## Abbreviations

AD: Atopic dermatitis
CMS: Dendritic core-multishell nanocarrier
CMS-ICC: CMS fluorescently labeled by covalently bound ICC
DAPI: 4′,6-Diamidino-2-phenylindole
FLIM: Fluorescence lifetime imaging microscopy
H&E: Hematoxylin and eosin
HPF: High power field
ICC: Indocarbocyanine
MPS: Mononuclear phagocytic system
OX: Oxazolone
TEWL: Transepidermal water loss

## References

[1] Boreham A, Pfaff M, Fleige E, Haag R, Alexiev U (2014). Nanodynamics of dendritic core-multishell nanocarriers. Langmuir.

[2] Radowski MR, Shukla A, von Berlepsch H, Böttcher C, Pickaert G, Rehage H (2007). Supramolecular aggregates of dendritic multishell architectures as universal nanocarriers. Angew Chem Int Ed.

[3] Lukowiak MC, Thota BNS, Haag R (2015). Dendritic core–shell systems as soft drug delivery nanocarriers. Biotechnol Adv.

[4] Rabe C, Fleige E, Vogtt K, Szekely N, Lindner P, Burchard W (2014). The multi-domain nanoparticle structure of a universal core-multi-shell nanocarrier. Polymer.

[5] Fleige E, Tyagi R, Haag R (2014). Dendronized core-multishell nanocarriers for solubilization of guest molecules. Nanocarriers.

[6] Küchler S, Abdel-Mottaleb M, Lamprecht A, Radowski MR, Haag R, Schäfer-Korting M (2009). Influence of nanocarrier type and size on skin delivery of hydrophilic agents. Int J Pharm.

[7] Küchler S, Radowski MR, Blaschke T, Dathe M, Plendl J, Haag R (2009). Nanoparticles for skin penetration enhancement—a comparison of a dendritic core-multishell-nanotransporter and solid lipid nanoparticles. Eur J Pharm Biopharm.

[8] Alnasif N, Zoschke C, Fleige E, Brodwolf R, Boreham A, Rühl E (2014). Penetration of normal, damaged and diseased skin—an in vitro study on dendritic core–multishell nanotransporters. J Controlled Release.

[9] SCCP (Scientific Commitee on Consumer Products of the European Commission) (2007) Safety of Nanomaterials in Cosmetic Producs; Available from: http://ec.europa.eu/health/ph_risk/committees/04_sccp/docs/sccp_o_123.pdf (as accessed on 2016.06.02)

[10] Odhiambo JA, Williams HC, Clayton TO, Robertson CF, Asher MI (2009). Global variations in prevalence of eczema symptoms in children from ISAAC Phase Three. J Allergy Clin Immunol.

[11] Bieber T (2010). Atopic dermatitis. Ann Dermatol.

[12] Bieber T (2008). Atopic dermatitis. N Engl J Med.

[13] Elias PM, Hatano Y, Williams ML (2008). Basis for the barrier abnormality in atopic dermatitis: outside-inside-outside pathogenic mechanisms. J Allergy Clin Immunol.

[14] Jensen JM, Fölster-Holst R, Baranowsky A, Schunck M, Winoto-Morbach S, Neumann C (2004). Impaired sphingomyelinase activity and epidermal differentiation in atopic dermatitis. J Invest Dermatol.

[15] Elias PM, Schmuth M (2009). Abnormal skin barrier in the etiopathogenesis of atopic dermatitis. Curr Opin Allergy Clin Immunol.

[16] Guttman-Yassky E, Suárez-Fariñas M, Chiricozzi A, Nograles KE, Shemer A, Fuentes-Duculan J (2009). Broad defects in epidermal cornification in atopic dermatitis identified through genomic analysis. J Allergy Clin Immunol.

[17] Proksch E, Brandner JM, Jensen J-M (2008). The skin: an indispensable barrier. Exp Dermatol.

[18] Jensen J-M, Pfeiffer S, Witt M, Bräutigam M, Neumann C, Weichenthal M (2009). Different effects of pimecrolimus and betamethasone on the skin barrier in patients with atopic dermatitis. J Allergy Clin Immunol.

[19] Ilves M, Palomäki J, Vippola M, Lehto M, Savolainen K, Savinko T, et al (2014) Topically applied ZnO nanoparticles suppress allergen induced skin inflammation but induce vigorous IgE production in the atopic dermatitis mouse model. Part. Fibre Toxicol 11(1). doi: 10.1186/s12989-014-0038-4 10.1186/s12989-014-0038-4 PMC4237966 25123235

[20] Man MQ, Hatano Y, Lee SH, Man M, Chang S, Feingold KR (2007). Characterization of a hapten-induced, murine model with multiple features of atopic dermatitis: structural, immunologic, and biochemical changes following single versus multiple oxazolone challenges. J Invest Dermatol.

[21] Boreham A, Kim T-Y, Spahn V, Stein C, Mundhenk L, Gruber AD (2011). Exploiting fluorescence lifetime plasticity in FLIM: target molecule localization in cells and tissues. ACS Med Chem Lett.

[22] Ostrowski A, Nordmeyer D, Boreham A, Brodwolf R, Mundhenk L, Fluhr JW (2014). Skin barrier disruptions in tape stripped and allergic dermatitis models have no effect on dermal penetration and systemic distribution of AHAPS-functionalized silica nanoparticles. Nanomedicine Nanotechnol Biol Med.

[23] Boreham A, Pikkemaat J, Volz P, Brodwolf R, Kuehne C, Licha K (2015). Detecting and quantifying biomolecular interactions of a dendritic polyglycerol sulfate nanoparticle using fluorescence lifetime measurements. Molecules.

[24] Kim T-Y, Winkler K, Alexiev U (2007). Picosecond multidimensional fluorescence spectroscopy: a tool to measure real-time protein dynamics during function. Photochem Photobiol.

[25] Ostrowski A, Nordmeyer D, Mundhenk L, Fluhr JW, Lademann J, Graf C (2014). AHAPS-functionalized silica nanoparticles do not modulate allergic contact dermatitis in mice. Nanoscale Res Lett.

[26] Kubo A, Nagao K, Yokouchi M, Sasaki H, Amagai M (2009). External antigen uptake by Langerhans cells with reorganization of epidermal tight junction barriers. J Exp Med.

[27] Simpson CL, Patel DM, Green KJ (2011). Deconstructing the skin: cytoarchitectural determinants of epidermal morphogenesis. Nat Rev Mol Cell Biol.

[28] Rouse JG, Yang J, Ryman-Rasmussen JP, Barron AR, Monteiro-Riviere NA (2007). Effects of mechanical flexion on the penetration of fullerene amino acid-derivatized peptide nanoparticles through skin. Nano Lett.

[29] Tinkle SS, Antonini JM, Rich BA, Roberts JR, Salmen R, Depree K (2003). Skin as a route of exposure and sensitization in hcronic beryllium disease. Environ Health Perspect.

[30] Lademann J, Knorr F, Richter H, Jung S, Meinke MC, Rühl E (2015). Hair follicles as a target structure for nanoparticles. J Innov Opt Health Sci.

[31] Boguniewicz M, Leung DYM (2011). Atopic dermatitis: a disease of altered skin barrier and immune dysregulation: Immune response in atopic dermatitis. Immunol Rev.

[32] Lin LL, Grice JE, Butler MK, Zvyagin AV, Becker W, Robertson TA (2011). Time-correlated single photon counting for simultaneous monitoring of zinc oxide nanoparticles and NAD(P)H in intact and barrier-disrupted volunteer skin. Pharm Res.

[33] Karande P, Jain A, Ergun K, Kispersky V, Mitragotri S (2005). Design principles of chemical penetration enhancers for transdermal drug delivery. Proc Natl Acad Sci.

[34] Prausnitz MR, Langer R (2008). Transdermal drug delivery. Nat Biotechnol.

[35] Pasparakis M, Haase I, Nestle FO (2014). Mechanisms regulating skin immunity and inflammation. Nat Rev Immunol.

[36] Godin B, Touitou E (2007). Transdermal skin delivery: predictions for humans from in vivo, ex vivo and animal models. Adv Drug Deliv Rev.

[37] Buckner JH (2010). Mechanisms of impaired regulation by CD4 + CD25 + FOXP3+ regulatory T cells in human autoimmune diseases. Nat Rev Immunol.

[38] Jin H, He R, Oyoshi M, Geha RS (2009). Animal models of atopic dermatitis. J Invest Dermatol.

[39] Fleige E, Achazi K, Schaletzki K, Triemer T, Haag R (2014). pH-responsive dendritic core–multishell nanocarriers. J Controlled Release.

[40] Treiber C, Quadir MA, Voigt P, Radowski M, Xu S, Munter L-M (2009). Cellular copper import by nanocarrier systems, intracellular availability, and effects on amyloid beta peptide secretion. Biochemistry (Mosc).

[41] Quadir MA, Radowski MR, Kratz F, Licha K, Hauff P, Haag R (2008). Dendritic multishell architectures for drug and dye transport. J Control Release.

