# Supplementary material for: Dendritic Core-Multishell Nanocarriers in Murine Models of Healthy and Atopic Skin
Source: Nanoscale Res Lett. 2017 Jan 23;12:64. doi: 10.1186/s11671-017-1835-0 (PMC5256633; doi:10.1186/s11671-017-1835-0)
Supplement: Additional file 1: — Detailed Methods. (DOCX 40.1 kb) [file 11671_2017_1835_MOESM1_ESM.docx]

**Supplemental Material**

**Dendritic Core-Multishell Nanocarriers in Murine Models of Healthy and Atopic Skin**

Moritz Radbruch, Hannah Pischon, Anja Ostrowski, Pierre Volz, Robert Brodwolf, Falko Neumann, Michael Unbehauen, Burkhard Kleuser, Rainer Haag, Nan Ma, Ulrike Alexiev, Lars Mundhenk, Achim D. Gruber

**Particle synthesis and characterization**

**Preparation of unlabeled CMS for *in vitro* toxicology**

CMS (DendroSol^®^, product number: 10-18-350) was purchased from Dendropharm, Berlin, Germany, and used as received.

**Preparation of indocarbocyanine labeled CMS for *in vivo* experiments**

CMS labled with the fluorescence dye indocarbocyanine were synthesized as described [1], with minor modifications. Briefly, a solution of hyperbranched polyglycerol amine (hPG-NH_2_, MW 10,000 g/mol, synthesized as previously published [2,3]) and *N*-hydroxysuccinimid (NHS)-activated ICC dye (Mivenion, Berlin, Germany) in methanol (Sigma Aldrich, Munich, Germany) was stirred for 6 hours at room temperature, followed by the addition of NHS-activated double-shell building block C_18_-mPEG_350_ (synthesized as described earlier [4]). After stirring for another 18 hours at room temperature, the crude product was purified using dialysis with a molecular weight cut-off of 2 kDa, benzoylated in methanol (Sigma Aldrich), yielding pure CMS-ICC (73% yield).

For further experiments, CMS were used in a 5 g/L, 0.9% sodium chloride solution [5].

**Characterization of indocarbocyanine labeled CMS for *in vivo* experiments**

Carriers were characterized using dynamic light scattering (DLS) and gel permeation chromatography (GPC) and resulted in values which were in accordance with previously published data [5].

The size of CMS-ICC was determined by DSL in a 5 g CMS /L 0.9% sodium chloride solution, using a Malvern Zetasizer Nano ZS (λ=633 nm; Malvern Instruments Ltd, Malvern, UK). In number-weighed size distribution the diameter of CMS-ICC was determined at 12.4 ± 3.5 nm.

GPC data were obtained using a Shimadzu (Japan) Liquid Chromatography setup with a PolarSil (PSS Polymer Standards Service GmbH, Germany) column (8 x 300 mm, 100 Å, 1000 Å, 3000 Å, 5 µm particle size). As eluent DMF (Promochem, Wesel, Germany; 0.3% LiBr, 0.6% acetic acid, Acros, Beel, Belgium) was used and the samples were measured against a polysterene calibration standard (PSS Polymer Standards Service GmbH, Germany). For CMS-ICC, the results found were: M_n_ = 40 kDa, M_w_ = 63 kDa, M_w_/M_n_ = 1.6. Measurement of unlabeled CMS resulted in M_n_ = 42 kDa and M_w_ = 77 kDa, M_w_/M_n_ = 1.8.

***In vivo* study**

**Animals**

Mice were purchased from Charles River (Sulzfeld, Germany) and allowed to settle in for one week prior to commencement of the study. They were housed in individually ventilated cages (21.5 ± 2°C; 45 ± 2% relative humidity, EU Norm II L) with a 12 h light/dark cycle (the light phase starting at 06:00 and ending 18:00). Mice had access to food and water ad libitum. Cages were covered with wood chips as bedding material, contained a mouse house (Tecniplast, Varese, Italy and Bioscape/Zoonlab, Castrop-Rauxel, Germany) and a tunnel. Soft paper and crushed cotton nesting material were available. All experiments were approved by the State Office of Health and Social Affairs, Berlin (LaGeSo G 0126/13) and performed according to German guidelines. During the entire experimental period, all animals were examined daily by a veterinarian.

**Induction of the atopic dermatitis model**

AD was induced via repeated topical hapten challenges with oxazolone (OX, Sigma-Aldrich, St. Louis, USA) in six- to eight-week-old, male, hairless SKH-1 mice (Charles River, Sulzfeld, Germany) as described [6] with minor modifications. Mice were sensitized with 50 µl of a 5% OX solution in acetone on a 1.5 x 1.5 cm area of the right flank on day -26. Immune response was allowed to develop for a week. Starting on day -19 repeated challenges with 60 µl 0.1% OX solution in ethanol were performed on the same area of the right flank every other day, continuing during the 5 days of treatment with test substance (day -19, -17, -15, -13, - 11, -9, -7, -5, -3, -1, 1 = second day of test substance application, 3 = fourth day of test substance application; i.e. twelve challenges altogether; see Figure S1 b). Challenges were performed approximately between 09:00 and 12:00, except challenges on day 1 and day 3, which were performed directly after the second round of test substance applications of on these days, i.e. approx. 17:00-18:00, see below.

**Topical application of nanocarriers**

CMS-ICC (44.5 µl, 5 g/L) in 0.9% sodium chloride (NaCl) solution (AlleMan Pharma GmbH, Rimbach, Germany) or the solvent alone were topically applied twice daily for 5 consecutive days to the right flank of healthy mice (n=3 for NaCL and CMS-ICC each) or the inflamed skin area of the atopic dermatitis model as described in Figure S1 a, b (n=3 for NaCl and n= 2 for CMS-ICC as one individual had to be excluded during the course of the study). Solutions were applied on the skin and then covered with a fine nylon mesh to assure even distribution of the aqueous liquids. The application side was carefully massaged for three minutes with a pipette tip with mesh in place. Substances were allowed to seep in for one hour prior to termination of the anesthesia (Isofluran CP®, cp-pharma, Burgdorf, Germany). The two daily applications of nanocarrier solution or vehicle were approximately 4 h apart. The first application each day was performed approximately between 08:30 - 13:00 each day, the second application approximately between 14:00 - 17:00.

**Subcutaneous injection of nanocarriers**

To mimic a complete transepidermal uptake, CMS-ICC in 0.9% NaCl solution (150 µl, 5 g/L, i.e. approx. 30 mg/kg body mass) or 0.9% NaCl solution alone were subcutaneously injected into the right flank of an additional group of SKH-1 mice twice daily for five consecutive days (Table 1, Figure S1 c). The first application each day was performed before noon, second application in the afternoon, approximately 4 h apart.

**Measurement of clinical parameters**

Transepidermal water loss (TEWL), erythema and hydration of the skin were measured using handheld probes (Tewameter® TM 300, Mexameter® MX 18 and Corneometer® CM 825 respectively, Courage and Khazaka electronic GmbH, Cologne, Germany). Erythema was measured photo-electronically using hemoglobins spectral absorption peak and skin hydration was estimated using the local capacity of the skin and outputting arbitrary “corneometer units” in these devices. The mexameter was operated using a magnet instead of standard pressure triggered operation. All clinical data were acquired daily immediately before the first application of test substances.

**Sampling and preparation of histological slides**

Immediately after euthanasia, tissues were either immersion fixed in 4% buffered formalin and later embedded in paraffin or submerged in -78°C methylbutane and stored at -80°C. Tissues were then processed for histology according to standard protocols [7].

**Measurement of epidermal thickness**

Hematoxylin and eosin staining (HE) was performed according to standard protocol on approx. 5 µm tissue sections. Epidermal thickness was measured as the mean of a total of 66 measurements per mouse. For this purpose, eleven measurements every 10 µm in three randomly selected areas between hair follicles were performed on two pieces of skin for each mouse, on a scanned HE slide (Slide Scanner Aperio CS2 and Aperio Image scope, version 10.2.2.2352, Leica Biosystems, Nussloch, Germany).

**Counting of lymphocytes, mast cells and eosinophils**

Immunohistochemistry for CD3 as surface marker of T-lymphocytes as well as staining and counting of mast cells and eosinophils were performed according to Ostrowski et al. [8]. T-lymphocytes were counted on scanned slides in 10 fields of maximal magnification (approx. 400 x 230 µm) in the epidermis and dermis separately. Mast cells and eosinophils were counted in 20 high power fields (400x magnification; BX41 microscope, Olympus, Japan) in the dermis only, as they do not usually move into the epidermis.

**Detection of ICC labeled CMS**

For fluorescence microscopy, slides were counterstained with 4′,6-diamidino-2-phenylindole (DAPI; Roti®-Mount FluorCare, Carl Roth, Karlsruhe, Germany) and distribution of CMS-NC was evaluated with a BX41 microscope (Olympus, Tokio, Japan) equipped with a digital camera (ColorView II, SIS, Münster, Germany; interface software: AnalySIS docu, version 5.0, SIS, Olympus, Tokio, Japan) and a mercury light source (U-LH100HG, Olympus, Tokyo, Japan).

***In vitro* toxicology**

**Cell culture conditions**

HaCat cells were cultured at 37*°*C*,* 5% CO_2_ concentration and 95% relative humidity. Cells were passaged when 70 % - 80 % confluence was reached.

**bis-AAF-R110 cytotoxicity assay**

Cells were seeded in a black 96 well plate with a density of 10,000 cells per well and cultured for 24 hours. The medium was removed and replaced with Medium containing CMS, followed by 24 hours of incubation. 20 µl of bis-alanyl-alanyl-phenylanlanyl-rhodamine 110 (bis-AAF-R110) substrate containing solution (Promega, Madison, USA) were added and incubated for 30 minutes. Ionomycin was used at 100 µM as a positive control due to its cytotoxicity. Untreated cells were used as a negative control. In this test, cells whose membrane integrity is compromised by cytotoxic substances release proteases which cleave rhodamine 110 from the substrate. Rhodamine fluorescence can then be measured in the medium. Fluorescence was measured at 485/520 nm using a Tecan Infinite 200 Pro microplate reader. Two independent experimental runs with five duplicates each were performed.

**Caspase-3/7 apoptose assay**

Cells were seeded in a transparent 96 well plate with a density of 10,000 cells per well and cultured for 24 hours. The medium was removed and replaced with medium containing CMS, followed by 24 hours of incubation. 100 µl of Caspase-Glo® 3/7 reagent (Promega, Madison, USA) was added to all wells, followed by 30 minutes of incubation at room temperature. Staurosporine was used as a positive control. Untreated cells were used as a negative control. This test contains a substrate that is cleaved by active caspase3/7 to aminoluciferin. This is then oxidized by luciferase, an enzyme also contained in the test reagent, causing luminescence. Luminescence was measured using a Tecan Infinite 200 Pro microplate reader. Three independent experimental runs with five duplicates each were performed.

**Cell counting kit-8 cell viability assay**

Cells were seeded in a transparent 96 well plate with a density of 10,000 cells per well and cultured for 24 hours. The medium was removed and replaced with medium containing CMS, followed by 24 hours of incubation. Subsequently, 10 µl of the pre-mixed Cell counting kit-8 (CCK-8) solution (Dojindo Molecular Technologies, Inc., Rockville, USA), containing the proprietary WST-8 tetrazolium salt, was added to each well. Viable cells reduce this salt to a formazan dye whose absorbance can be measured in the medium. Absorbance was measured at 450 nm using a Tecan Infinite 200 Pro microplate reader after two hours. Two independent experimental runs with five duplicates each were performed.

**H2-DCF-DA assay for reactive oxygen species**

Cells were seeded in a black 96 well plate with a density of 10,000 cells per well and cultured for 24 hours. The medium was removed and replaced with PBS containing CMS or H_2_O_2_ (positive control) for one hour. Untreated cells were used as a negative control. After a washing step 2',7'-dichlordihydrofluorescein-diacetat (H_2_DCF-DA), purchased from Invitrogen™ (Life Technologies), diluted in PBS with glucose in a final concentration of 20 µM was added and incubated for another hour. In the presence of reactive oxygen species, strongly fluorescing 2',7'-dichlorfluorescein (DCF) is formed from H_2_DCF-DA. Fluorescence was then detected using a Tecan Infinite 200 Pro microplate reader at wavelengths for excitation/emission of 485 nm/520 nm. Two independent experimental runs with five duplicates each were performed.

**References:**

1. Boreham A, Pfaff M, Fleige E, Haag R, Alexiev U. Nanodynamics of dendritic core-multishell nanocarriers. Langmuir. 2014;30:1686–95.

2. Roller S, Zhou H, Haag R. High-loading polyglycerol supported reagents for Mitsunobu- and acylation-reactions and other useful polyglycerol derivatives. Mol. Divers. 2005;9:305–16.

3. Sunder A, Hanselmann R, Frey H, Mülhaupt R. Controlled Synthesis of Hyperbranched Polyglycerols by Ring-Opening Multibranching Polymerization. Macromolecules. 1999;32:4240–6.

4. Radowski MR, Shukla A, von Berlepsch H, Böttcher C, Pickaert G, Rehage H, et al. Supramolecular aggregates of dendritic multishell architectures as universal nanocarriers. Angew. Chem. Int. Ed Engl. 2007;46:1265–9.

5. Küchler S, Radowski MR, Blaschke T, Dathe M, Plendl J, Haag R, et al. Nanoparticles for skin penetration enhancement – A comparison of a dendritic core-multishell-nanotransporter and solid lipid nanoparticles. Eur. J. Pharm. Biopharm. 2009;71:243–50.

6. Man M-Q, Hatano Y, Lee SH, Man M, Chang S, Feingold KR, et al. Characterization of a Hapten-Induced, Murine Model with Multiple Features of Atopic Dermatitis: Structural, Immunologic, and Biochemical Changes following Single Versus Multiple Oxazolone Challenges. J. Invest. Dermatol. 2007;128:79–86.

7. Ostrowski A, Nordmeyer D, Boreham A, Brodwolf R, Mundhenk L, Fluhr JW, et al. Skin barrier disruptions in tape stripped and allergic dermatitis models have no effect on dermal penetration and systemic distribution of AHAPS-functionalized silica nanoparticles. Nanomedicine. 2014;10:1571–81.

8. Ostrowski A, Nordmeyer D, Mundhenk L, Fluhr JW, Lademann J, Graf C, et al. AHAPS-functionalized silica nanoparticles do not modulate allergic contact dermatitis in mice. Nanoscale Res. Lett. 2014;9:524.
