# Supplementary figures and images for: Dendritic Core-Multishell Nanocarriers in Murine Models of Healthy and Atopic Skin
Source: Nanoscale Res Lett. 2017 Jan 23;12:64. doi: 10.1186/s11671-017-1835-0 (PMC5256633; doi:10.1186/s11671-017-1835-0)

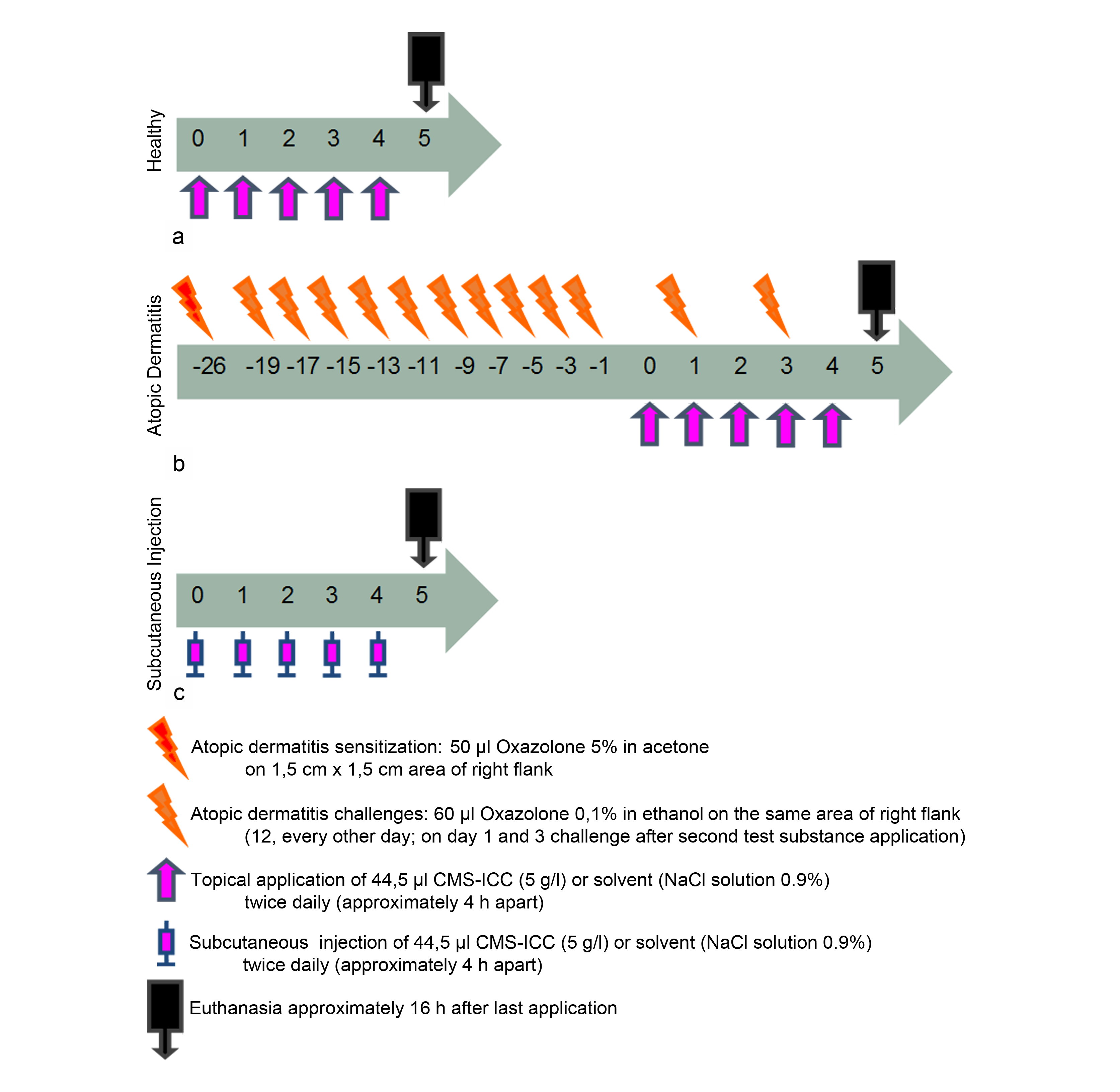

Supplement: Additional file 2: — Schematic Experimental Protocol. (TIFF 916 kb) [file 11671_2017_1835_MOESM2_ESM.tif]
